# Point of Care Ultrasound Diagnosis of Upper Gastrointestinal Bleeding

**DOI:** 10.7759/cureus.1956

**Published:** 2017-12-17

**Authors:** Robert P Jamplis, Lucas Friedman, Srikar Adhikari

**Affiliations:** 1 College of Medicine, University of Arizona College of Medicine-Tucson; 2 Department of Emergency Medicine at Riverside Community Hospital, University of California, Riverside; 3 Department of Emergency Medicine, University of Arizona

**Keywords:** focused assessment with sonography for trauma, point-of-care ultrasound, pocus, bedside ultrasound, ultrasound, upper gastrointestinal bleed, gastric contents, esophageal varices, gastric ultrasound, hypotension

## Abstract

A 28-year-old male was brought to the emergency department by the Emergency medical services (EMS) after being found unconscious and unresponsive. Upon arrival, he was hypotensive, intubated with a Glasgow Coma Scale (GCS) 3T, without the signs of trauma or the evidence of bleeding. A focused assessment with sonography in trauma (FAST), point-of-care ultrasound (POCUS) was performed, obscuring part of the spleen from the distended stomach, which was filled with the heterogeneous contents, with the internal movement being identified. This was found to be blood after orogastric (OG) tube was placed for suction. The bedside endoscopy confirmed active variceal bleeding. This case illustrates the potential utility of the ultrasound in detecting the upper gastrointestinal bleeding.

## Introduction

Upper gastrointestinal bleeding (UGIB) is commonly presented to the emergency department with an estimated incidence rate of 50-100 per 100,000 and more than 20,000 deaths annually in the United States [1]. The major causes of UGIB include peptic ulcer disease, esophageal varices, and acute hemorrhagic gastritis [2]. The acute (overt) bleeds present with hematemesis, melena, or hematochezia, occult (chronic) bleeds are identified by a positive fecal occult blood, and in obscure bleeds by the endoscopy [3]. Massive gastrointestinal (GI) hemorrhaging can also present with symptoms of hemodynamic instability such as syncope, postural hypotension, tachycardia, and shock [4].

The primary investigative procedure for the diagnosis of UGIB is the upper endoscopy, which has a sensitivity and specificity of 92-98% and 30-100% respectively [5]. The additional diagnostic imaging includes the computed tomography (CT) angiography, the catheter angiography and, the nuclear scintigraphy. Currently, there is scant literature exploring the use of point-of-care ultrasound (POCUS) in diagnosing UGIB.

## Case presentation

A 28-year-old male, who had a past medical history of cirrhosis and ethanol (EtOH) abuse, was brought to the emergency department after being found unconscious and unresponsive in the bed. The patient was defibrillated initially and was then noted by EMS to be in pulseless electrical activity (PEA). Return of spontaneous circulation (ROSC) was achieved after four rounds of epinephrine and cardiopulmonary resuscitation (CPR).

Upon arrival to the emergency department, the patient was bradycardic, hypotensive, intubated with a GCS of 3T, without signs of trauma or evidence of bleeding. The patient was given intravenous (IV) fluids, 2 units packed red blood cells (PRBC), placed on epinephrine and vasopressin drips, and given a dose of corticosteroids.

**Figure 1:**
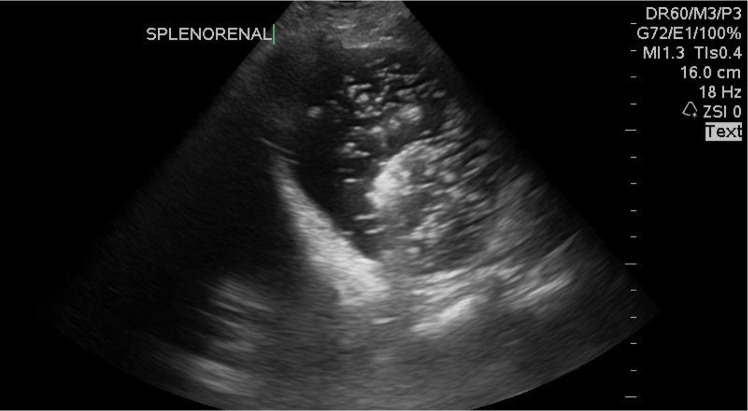
The distended stomach with the heterogeneous internal echogenicity.

The focused assessment with sonography (FAST) was performed and revealed a distended stomach filled with heterogeneous contents, obscuring part of the spleen (Figure 1, Video 1 and Video 2)

**Video 1:** The distended stomach with the heterogeneous internal echogenicity.

**Video 2:** The distended stomach with the heterogeneous internal echogenicity.

The blood was monitored upon placing an orogastric (OG) tube to suction, and the stomach could be seen decompressing on the ultrasound, with an area of the internal movement that may have represented the active extravasation (See Video 3).

**Video 3:** The distended stomach with the internal movement.

**Figure 2:**
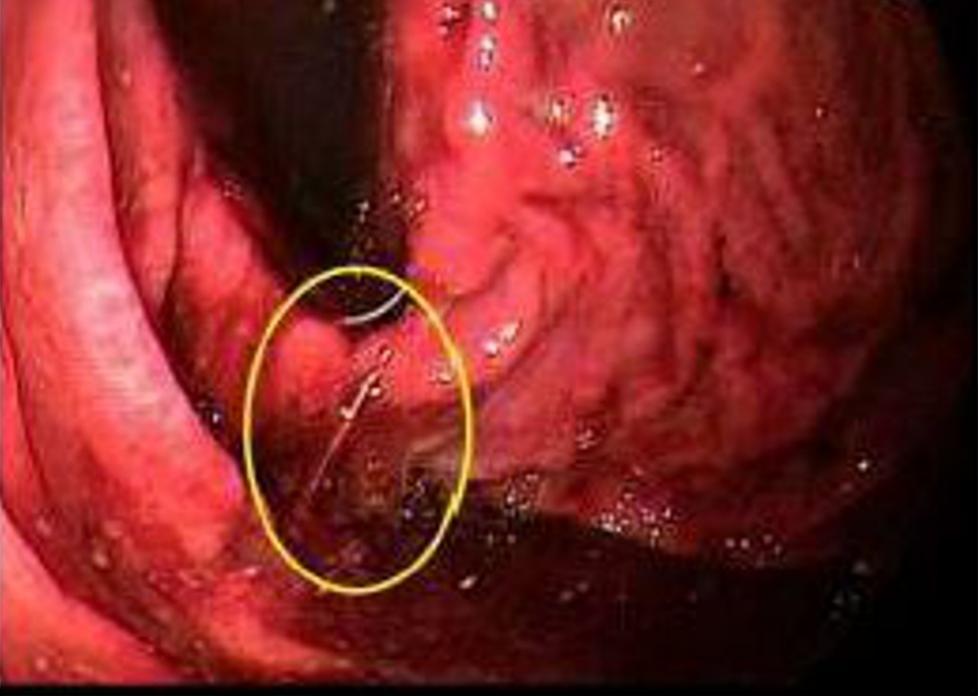
The active extravasation on the endoscopy.

The proton-pump inhibitors (PPI) and octreotide drips were started, and a massive transfusion protocol was initiated (including a total of 14 units of packed red blood cells). The bedside endoscopy was performed confirming active variceal bleeding at the gastroesophageal junction, which was banded with hemostasis (See Figure 2).

The patient was resuscitated with the massive transfusion protocol and admitted to the intensive care unit (ICU). Unfortunately, the patient remained unresponsive and the family withdrew care and the patient died from hypoxic injuries.

## Discussion

Historically, the gastric ultrasonography was used primarily to diagnose the lesions in the gastric wall and to evaluate the gastric motility and emptying [6]. However, because of its availability and non-invasive nature, the gastric ultrasound is used to determine the gastric content and the volume. Both gastric content and the volume are associated with the perioperative aspiration risk, a rare but serious complication of anesthesia [7]. The studies have demonstrated that the various contents of the stomach (gas, fluid, and solid) can be differentiated based on their appearance on the ultrasound. For example, the baseline gastric secretions and water appear hypoechoic or anechoic, the gas bubbles appear as multiple freely moving echoes, thick fluids have increased the echogenicity and solid-fluid mixes with air during chewing to produce ringing artifacts that create a “starry night” like appearance [6]. Additionally, the measurements of the stomach diameter can be taken to estimate the gastric volume, which is directly related to the risk of aspiration [7]. Consequently, the gastric ultrasound is now being used to evaluate the risk of aspiration in the perioperative environment and to guide the anesthetic management using a clinical algorithm (empty = low risk; solid = high risk; fluid = assess volume to determine risk).

A standard scanning technique involves using a low frequency, curvilinear transducer, placed in a sagittal plane at the epigastrium and then sweeping from the left to the right subcostal margins [8]. Moving the patient from the supine to a right lateral decubitus position may further aid in visualizing the gastric contents as they fill the antrum. In the exact clinical scenario, a distended stomach with heterogenous contents may represent hemorrhage with the clot and the active movement may also represent active extravasation.

## Conclusions

Similar to its adoption as an assessment tool for the aspiration risk, this case demonstrates the potential use of the gastric ultrasound as a screening tool for UGIB in an undifferentiated hypotensive patient. In conjunction with a POCUS screening protocol, such as the Rapid Ultrasound for Shock and Hypotension (RUSH) protocol, the gastric ultrasound also has the potential to detect a UGIB early, as in this case, prompting the placement of an OG tube. Further studies are required to confirm the utility of the ultrasound.
